# Correction to “Bronchiolar Adenoma/Ciliated Muconodular Papillary Tumour With a 17‐Year Follow‐Up: A Case Report”

**DOI:** 10.1002/rcr2.70351

**Published:** 2025-09-25

Y. Hayashi, E. Ando, Y. Matsuo, et al., “Bronchiolar Adenoma/Ciliated Muconodular Papillary Tumour With a 17‐Year Follow‐Up: A Case Report,” *Respirology Case Reports* 13, no. 8 (2025): e70337, https://doi.org/10.1002/rcr2.70337.

Figure 1 caption is incorrect. It should be corrected as follows:

Chest CT images. (a) A 24‐mm solid nodule in the same location, showing slow progression over 17 years (2024, kilovoltage peak; 120 kV, milliampere; auto mA, rotation time; 0.5 s, slice thickness; 5 mm slice). (b) A 13‐mm nodule in the right middle lobe (2007, kilovoltage peak; 120 kV, milliampere; 200 mA, rotation time; 1.0 s, slice thickness; 10 mm slice).

We apologise for this error.

